# The Golgi Calcium ATPase Pump Plays an Essential Role in Adeno-associated Virus Trafficking and Transduction

**DOI:** 10.1128/JVI.01604-20

**Published:** 2020-10-14

**Authors:** Victoria J. Madigan, Garrett E. Berry, Tyne O. Tyson, Dasean Nardone-White, Jonathan Ark, Zachary C. Elmore, Giridhar Murlidharan, Heather A. Vincent, Aravind Asokan

**Affiliations:** a Curriculum in Genetics and Molecular Biology, University of North Carolina at Chapel Hill, Chapel Hill, North Carolina, USA; b Department of Surgery, Duke University School of Medicine, Durham, North Carolina, USA; c Department of Molecular Genetics & Microbiology, Duke University School of Medicine, Durham, North Carolina, USA; d Department of Biomedical Engineering, Regeneration Next, Duke University, Durham, North Carolina, USA; University of California, Irvine

**Keywords:** adeno-associated virus, calcium flux, gene therapy, subcellular localization, vesicular trafficking

## Abstract

Adeno-associated viruses (AAVs) are dependoparvoviruses that have proven useful for therapeutic gene transfer; however, our understanding of host factors that influence AAV trafficking and transduction is still evolving. Here, we investigated the role of cellular calcium in the AAV infectious pathway. First, we demonstrated a critical role for the host Golgi compartment-resident ATP-powered calcium pump (secretory pathway calcium ATPase 1 [SPCA1]) encoded by the *ATP2C1* gene in AAV infection. CRISPR-based knockout (KO) of *ATP2C1* decreases transduction by different AAV serotypes. *ATP2C1* KO does not appear to inhibit AAV binding, cellular uptake, or nuclear entry; however, capsids within *ATP2C1* KO cells demonstrate dispersed and punctate trafficking distinct from the perinuclear, *trans*-Golgi pattern observed in normal cells. In addition, we observed a defect in the ability of AAV capsids to undergo conformational changes and support efficient vector genome transcription in *ATP2C1* KO cells. The calcium chelator BAPTA-AM, which reduces cytosolic calcium, rescues the defective *ATP2C1* KO phenotype and AAV transduction *in vitro*. Conversely, the calcium ionophore ionomycin, which disrupts calcium gradients, blocks AAV transduction. Further, we demonstrated that modulating calcium in the murine brain using BAPTA-AM augments AAV gene expression *in vivo*. Taking these data together, we postulate that the maintenance of an intracellular calcium gradient by the calcium ATPase and processing within the Golgi compartment are essential for priming the capsid to support efficient AAV genome transcription.

**IMPORTANCE** Adeno-associated viruses (AAVs) have proven to be effective gene transfer vectors. However, our understanding of how the host cell environment influences AAV transduction is still evolving. In the present study, we investigated the role of *ATP2C1*, which encodes a membrane calcium transport pump, SPCA1, essential for maintaining cellular calcium homeostasis on AAV transduction. Our results indicate that cellular calcium is essential for efficient intracellular trafficking and conformational changes in the AAV capsid that support efficient genome transcription. Further, we show that pharmacological modulation of cellular calcium levels can potentially be applied to improve the AAV gene transfer efficiency.

## Abstract (precis)

Adeno-associated viruses (AAVs) have proven to be effective gene transfer vectors. However, our understanding of how the host cell environment influences AAV transduction is still evolving. In the present study, we investigated the role of *ATP2C1*, which encodes a membrane calcium transport pump, SPCA1, essential for maintaining cellular calcium homeostasis on AAV transduction. Our results indicate that cellular calcium is essential for efficient intracellular trafficking and conformational changes in the AAV capsid that support efficient genome transcription. Further, we show that pharmacological modulation of cellular calcium levels can potentially be applied to improve the AAV gene transfer efficiency.

## INTRODUCTION

Adeno-associated viruses (AAVs) are helper-dependent parvoviruses that have been successfully utilized for therapeutic gene transfer (1, 2). Wild-type AAVs package a single-stranded DNA genome containing two open reading frames, *Rep* and *Cap*, flanked by inverted terminal repeats (ITRs). While *Rep* carries the genes necessary for AAV replication, *Cap* encodes the capsid structural proteins VP1, VP2, and VP3, the assembly-activating protein (AAP), and the recently discovered membrane-associated accessory protein (MAAP) (3, 4). The VP1, VP2, and VP3 subunits assemble into icosahedral capsids in an approximately 1:1:10 ratio. While VP1 and VP2 are the less abundant proteins in the AAV capsid, they provide key functions essential for AAV infectivity, including putative nuclear localization signals and a buried phospholipase domain, which becomes externalized as the capsid traffics through intracellular environments (5–8).

Although different AAV serotypes appear to utilize various cell surface glycans as attachment factors, several common host factors identified in a haploid genetic screen, such as AAVR (KIAA0319L), GPR108, and RNF121, have been shown to be essential for infection (9). Of note, AAVR and GPR108 both influence AAV trafficking through the Golgi network (9, 10). In this regard, an interesting gene listed as a hit in the aforementioned screen is *ATP2C1* (9). The *ATP2C1* gene, which encodes the Golgi compartment-resident ATP-powered calcium pump SPCA1, is critical for regulating Golgi compartment calcium content and maintaining the ultrastructure of this subcellular organelle and for functions such as sorting of proteins to the plasma membrane (11). In humans, mutations in *ATP2C1* have been associated with Hailey-Hailey disease, which causes dysfunction in keratinocyte Ca^2+^ storage, resulting in fragility and blistering of the skin (12).

In the current study, we investigated the role of *ATP2C1* and calcium homeostasis in AAV transduction. We generated a clonal CRISPR knockout (KO) line for *ATP2C1* and demonstrated that AAV transduction in this cell line is reduced. We then employed a battery of cell-based assays probing AAV trafficking, and we show that viral binding, uptake, and nuclear association are not compromised in *ATP2C1* KO cells. However, immunofluorescent staining of intact AAV capsids revealed altered intracellular trafficking of *ATP2C1* KO cells relative to control cells. Further analysis showed that capsids in *ATP2C1* KO cells are defective in conformational changes essential to support AAV genome transcription. We then utilized pharmacological modulation to support these observations and further shed light on the interplay between cellular calcium gradients and AAV transduction. Last, we exploited our learnings to pharmacologically augment AAV transduction in the mouse brain *in vivo*.

## RESULTS

### ATP2C1 is an essential host factor for AAV transduction.

**FIG 1:**
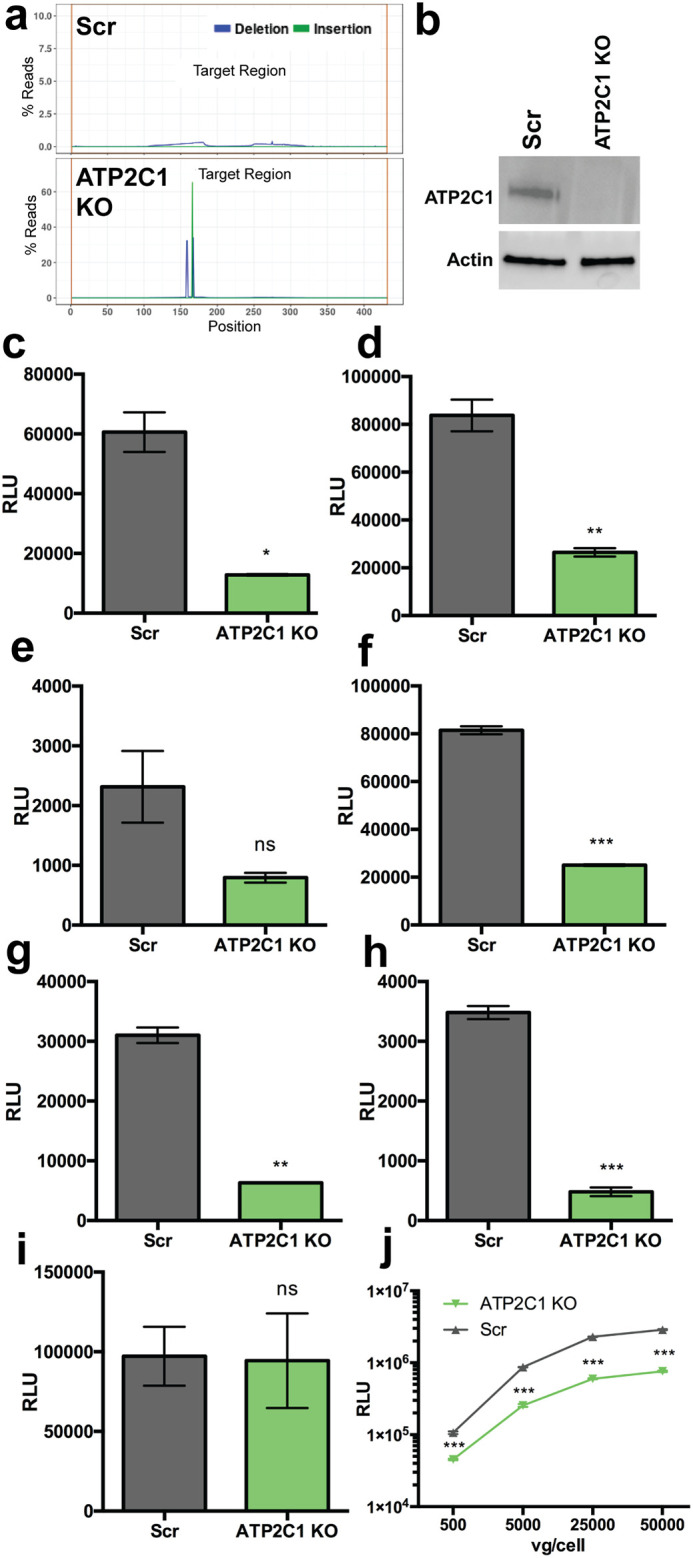
*ATP2C1* KO decreases AAV transduction efficiency. (a) Mutation rates of a target indel region in Scr and *ATP2C1* KO cell lines. (b) Western blot of lysates from Scr and ATP2C1 KO cell lines probed with anti-ATP2C1 and anti-actin antibodies. (c to h) AAV1 at 10,000 vg/cell (c), AAV2 at 2,000 vg/cell (d), AAV4 at 20,000 vg/cell (e), AAV6 at 10,000 vg/cell (f), AAV8 at 20,000 vg/cell (g), and AAV9 at 20,000 vg/cell (h) packaging CBA-luciferase. (i) Transfection of the pTR-CBA-luciferase genome. (j) Dose curve of AAV2-luciferase on Scr and ATP2C1 KO Huh7 cells. *, *P < *0.05; **, *P < *0.01; ***, *P < *0.005; ns, not significant.

To investigate the potential role of *ATP2C1* in AAV transduction, we derived a clonal CRISPR knockout cell line in Huh7 hepatocarcinoma cells. Briefly, guide RNAs against *ATP2C1* were cloned into the LentiCRISPRv2 backbone, and recombinant lentivirus in which this cassette was packaged was used to transduce Huh7 cells. A clonal knockout population was isolated via serial dilution and subjected to high-throughput amplicon sequencing to assess indels in the *ATP2C1* gene relative to Scr control cells (Fig. 1A). Amplicon sequencing confirmed mutation of *ATP2C1* reads in the clonal KO line, with deletions and insertions spanning the CRISPR indel site (Fig. 1a). Furthermore, loss of ATP2C1 protein expression was confirmed through Western blotting in *ATP2C1* KO cells (Fig. 1b).

*ATP2C1* KO cells were then transduced alongside a scramble (Scr) nontargeting guide control cells with different AAV serotypes packaging a chicken β-actin (CBA)-luciferase transgene, including AAV1, AAV2, AAV4, AAV6, AAV8, and AAV9. Luciferase expression was quantified via luminometer to determine relative transgene expression, and *ATP2C1* KO cells demonstrated reduced transduction with all serotypes tested (Fig. 1c to h). There was no significant change in luciferase expression upon transfecting the AAV vector genome carrying the plasmid construct (Fig. 1i), suggesting that *ATP2C1* expression does not directly regulate AAV genome transcription or transgene expression. Scr and *ATP2C1* KO cells were also transduced with AAV2-luciferase at a range of doses, with the knockout demonstrating reduced transduction regardless of the quantity of viral genomes added per cell (Fig. 1j). Thus, *ATP2C1* is necessary for AAV transduction, regardless of serotype or dose.

### ATP2C1 KO results in altered intracellular trafficking and decreased AAV genome transcription.

**FIG 2:**
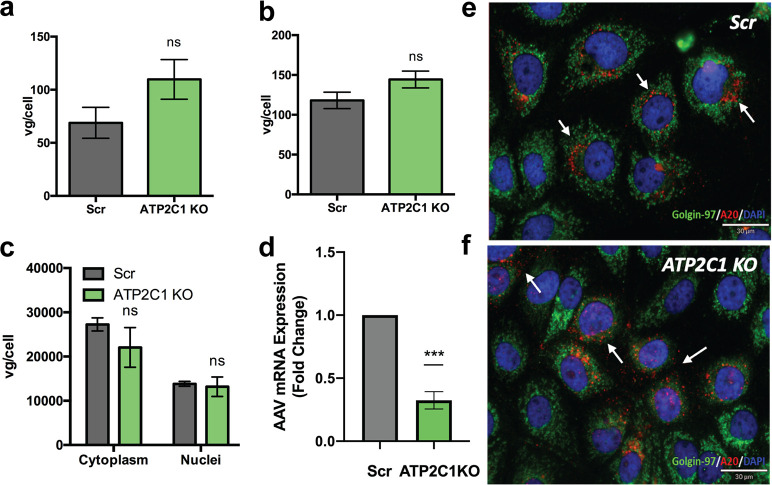
*ATP2C1* KO adversely affects AAV perinuclear localization and mRNA transcription. (a and b) Binding (a) and uptake (b) of AAV2 on Scr and *ATP2C1* KO Huh7. (c) Nuclear fractionation of Scr and *ATP2C1* KO Huh7 cells 18 h after transduction with AAV2-luciferase followed by qPCR to assess localization of viral genomes. (d) Relative levels of mRNA transcribed from AAV vector genomes. (e and f) Differential perinuclear versus dispersed, punctate A20 staining (red) of AAV2 capsids (white arrows) trafficking through Scr (e) and *ATP2C1* KO (f) cells at 18 h postransduction and the golgin-97 marker (green) for the Golgi compartment. **, *P < *0.01; ns, not significant.

We probed multiple steps of the AAV infectious cycle in Scr and *ATP2C1* KO cells. Cellular binding and uptake of AAV2 capsids were unaffected by the absence of *ATP2C1* (Fig. 2a and b). To interrogate nuclear association of AAV virions, Scr and *ATP2C1* KO cells were transduced for 18 h with AAV2-luciferase and then subjected to nuclear fractionation and quantitative PCR (qPCR) to determine the localization of viral genomes. Scr and *ATP2C1* KO cells did not demonstrate any significant difference in the quantity of cytosolic or nuclear virions (Fig. 2c). However, we observed a statistically significant reduction (∼3-fold) in the amount of mRNA transcribed from AAV vector genomes, consistent with the decreased transduction observed earlier in *ATP2C1* KO cells compared to Scr controls (Fig. 2d).

We then utilized confocal microscopy to visualize intracellular trafficking patterns of AAV capsids in the presence and absence of *ATP2C1*. Briefly, Scr and *ATP2C1* KO cells were transduced with AAV2 for 18 h and then subjected to immunofluorescent staining with the A20 antibody, which recognizes intact AAV2 capsids in their native conformation (13). Overall, Scr and *ATP2C1* KO cells displayed similar signal intensities with A20, consistent with qPCR data suggesting that viral entry into the host cell is unaffected (Fig. 2e and f). However, while Scr cells displayed perinuclear staining of intact virions aligned with the golgin-97 marker found on the Golgi compartment, *ATP2C1* KO cells showed a more dispersed and punctate pattern of intact AAV virions throughout the cytosol (Fig. 2e and f). These results implied a potential defect in intracellular transport of AAV particles into the perinuclear Golgi compartment.

### AAV capsids show conformational defects while trafficking in *ATP2C1* KO cells.

**FIG 3:**
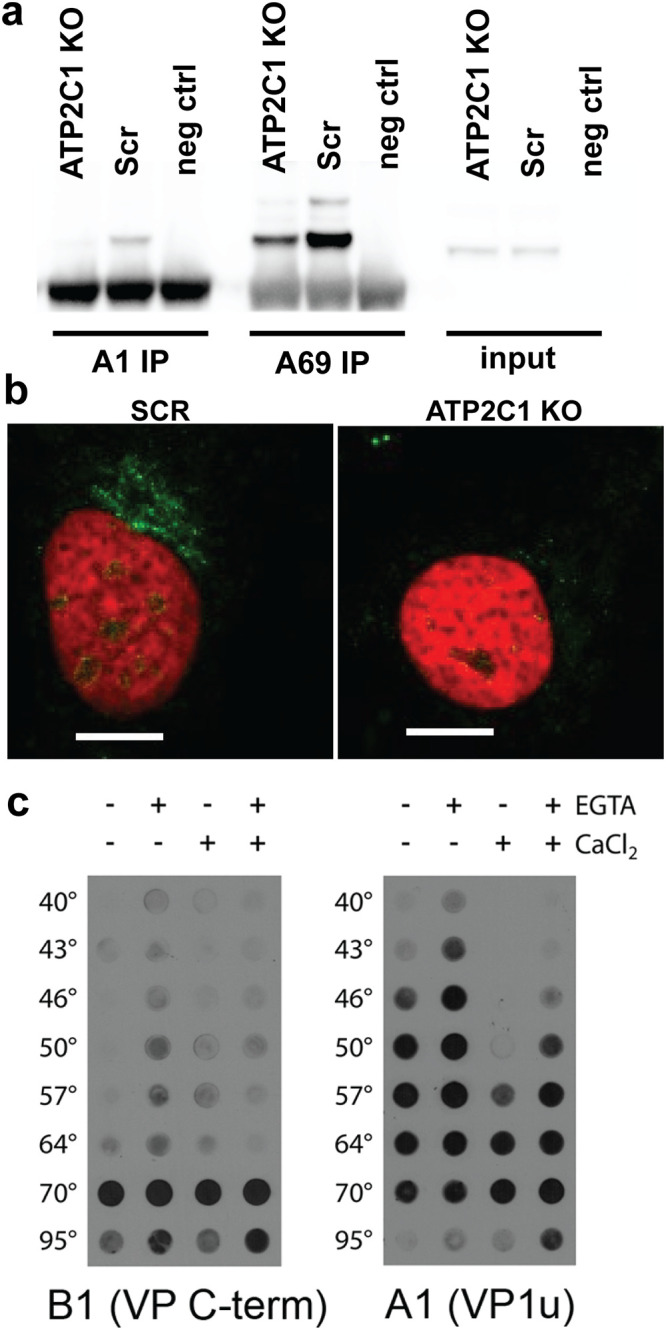
*ATP2C1* KO and calcium levels influence capsid conformational changes. (a) Immunoprecipitation of VP1 (A1) and VP1/VP2 (A69) of lysate from Scr and *ATP2C1* KO Huh7 cells 18 h after transduction with AAV2 luciferase. (b) Confocal immunofluorescence microscopy of Scr and *ATP2C1* KO Huh7 cells stained for externalized VP1/VP2 (A69). Bars, 10 μm. (c) Native dot blots with the B1 monoclonal antibody detecting the VP1 C-terminal domain and A1 monoclonal antibody detecting the thermally induced externalization of VP1 N termini from within the AAV capsid. Capsid stability assays were carried out using a thermal cycler with different AAV samples in the presence of the calcium chelator EGTA and/or extraneously added calcium ions.

To understand the trafficking defect observed with AAV particles, we investigated AAV capsid dynamics during infection of Scr and *ATP2C1* KO cells. Scr and *ATP2C1* KO cells were transduced with AAV2 for 18 h and subjected to immunoprecipitation (IP) under nondenaturing conditions with A69 or A1 monoclonal antibodies. The latter are known to bind linear epitopes located in N-terminal domains of VP1 plus VP2 and of VP1 only, respectively (Fig. 3a). These capsid epitopes are recognized by antibodies only when the otherwise buried VP1/VP2 and VP1 N-terminal domains are externalized, causing conformational changes in the capsid. In contrast, the monoclonal antibody B1 binds a linear C-terminal epitope exposed in all denatured VPs (14, 15). While Scr and *ATP2C1* KO cells had the same B1 signal in input lysate, IP with A69 as well as A1 antibodies pulled down reduced levels of AAV VPs from *ATP2C1* KO cell lysate relative to Scr controls (Fig. 3a). These data suggest that at 18 h postransduction, considerably fewer capsids have undergone conformational changes in *ATP2C1* KO cells than in Scr control cells. This observation is also consistent with confocal immunofluorescence microscopy studies, wherein AAV capsids stained with the A69 antibody appear to have undergone conformational changes in the perinuclear region of Scr control cells, but not in *ATP2C1* KO cells, at 18 h postransduction (Fig. 3b).

Since *ATP2C1*-encoded SPCA1 regulates calcium transport from the cytosol to the Golgi compartment, we hypothesized that altered calcium levels could affect AAV capsid dynamics during trafficking. To directly assess the impact of calcium on capsid conformational changes, we interrogated the impact of calcium on VP1 N-terminal domain externalization in a cell-free capsid thermal stability assay (16). Briefly, we utilized a thermal cycler to enable heat-induced capsid conformational changes in the presence or absence of calcium and/or the calcium chelator EGTA. The virus was then subjected to immunoblot analysis using the VP1 N-terminal specific antibody A1 described above. As observed in Fig. 3c, conformational changes were observed at 50°C in phosphate-buffered saline when no EGTA or calcium was added. However, treatment of capsids with EGTA caused exposure of the A1 epitope at a lower temperature (46°C). Furthermore, treatment of capsids with calcium ions shifted the conformational changes to a higher transition temperature (64°C). These results demonstrate that calcium can influence capsid conformational dynamics that likely occur along with externalization of the VP1 N-terminal phospholipase A2 (PLA2) domain, which has been shown previously to be essential for productive AAV infection (5). It is important to note that epitopes recognized by A69 and A1 antibodies are located toward the C-terminal end of the PLA2 domain and as such do not report PLA2 domain externalization or enzyme activity. Rather, the decreased antibody recognition signal suggests defects in capsid conformational changes accompanying PLA2 externalization.

### Pharmacological modulation of cellular calcium can regulate AAV transduction in a cell-specific manner.

**FIG 4:**
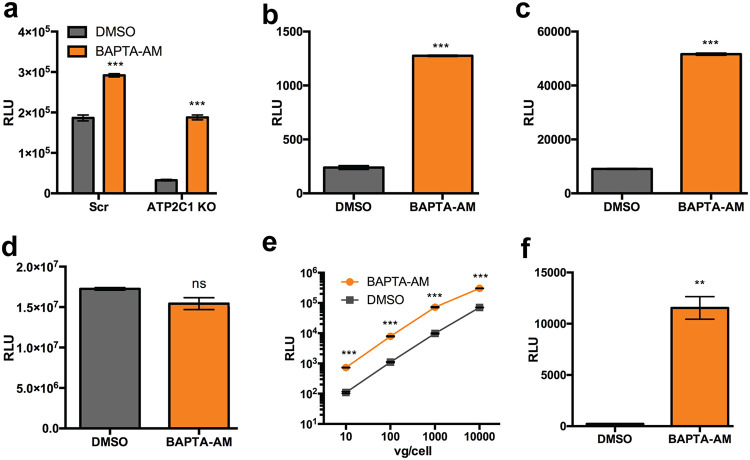
The intracellular calcium-chelating agent BAPTA-AM enhances AAV transduction. (a) AAV2 luciferase transduction of Scr and *ATP2C1* KO Huh7 cells following treatment with BAPTA-AM (8 μM). (b and c) Luciferase reporter expression in HeLa cells pretreated with dimethyl sulfoxide (DMSO) or BAPTA-AM (20 μM) and infected with AAV1 luciferase (b) and AAV2 luciferase (c). (d) Transfection of HeLa cells treated with BAPTA-AM or DMSO control with pTR-CBA-luciferase. (e) HeLa cells pretreated with either DMSO control or BAPTA-AM and infected with AAV2-Luc at various vector doses. (f) Luciferase reporter expression in MB114 cells pretreated with DMSO or BAPTA-AM (20 μM) and infected with AAV2-luciferase. **, *P < *0.01; ***, *P < *0.005; ns, not significant.

We then investigated whether pharmacologic modulation of calcium gradients can influence AAV transduction in Scr and *ATP2C1* KO cells. BAPTA-AM [1,2-bis-(2-aminophenoxy)ethane-*N*,*N*,*N*′,*N*′-tetraacetic acid tetra(acetoxymethyl) ester] is a highly selective, cell-permeative calcium chelator which serves to decrease cytosolic calcium, thus intensifying intracellular gradients between the cytosol and subcellular compartments. Once BAPTA-AM enters the cell, cytosolic esterases cleave off the acetoxymethyl (AM) ester, activating the chelator and trapping the molecule in the chelated form within the cytosol. In Huh7 cells, BAPTA-AM treatment enhanced AAV2 transduction marginally (∼1.5-fold) in Scr cells but enhanced transduction significantly (nearly 6-fold) in *ATP2C1* KO cells (Fig. 4a). Further, BAPTA-AM treatment increased both AAV1 and AAV2 transduction by >5-fold in HeLa cells (Fig. 4b and c). However, transfection of the pTR-CBA-luciferase AAV genome was not affected by BAPTA-AM treatment (Fig. 4D). Transduction of HeLa cells with a range of doses of AAV2-luciferase demonstrated that BAPTA-AM treatment augmented AAV transduction regardless of dose (Fig. 4e). Further, treatment of a murine endothelial cell line MB114 with BAPTA-AM resulted in a >50-fold increase in AAV2 transduction (Fig. 4f). These results demonstrate that cytosolic calcium chelation is a conserved mechanism to enhance AAV transduction.

**FIG 5:**
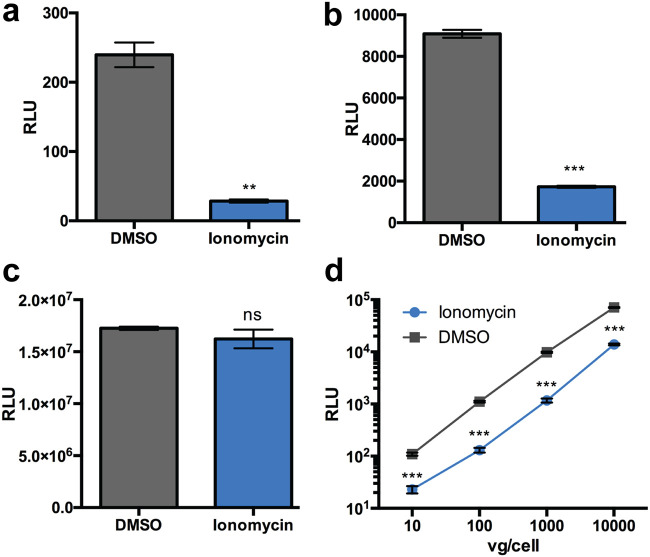
The calcium ionophore ionomycin blocks AAV transduction. (a and b) Luciferase reporter expression in HeLa cells pretreated with DMSO or ionomycin (10 μM) infected with AAV1 luciferase (a) and AAV2-luciferase (b). (c) Transfection of HeLa cells treated with ionomycin or DMSO control with pTR-CBA-luciferase. (d) HeLa cells pretreated with either DMSO or ionomycin and infected with AAV2-Luc at various vector doses. **, *P < *0.01; ***, *P < *0.005; ns, not significant.

To extend these findings, we evaluated the impact of ionomycin, an ionophore that allows the flow of calcium across biological membranes by creating channels, thereby disrupting extracellular-to-intracellular calcium gradients (17). Additionally, ionomycin is known to promote release of calcium from subcellular compartments such as the endoplasmic reticulum (ER) into the cytosol (18). Treatment with ionomycin decreased transduction by 5- to 10-fold in HeLa cells with AAV1 and AAV2 (Fig. 5a and b). However, ionomycin treatment did not affect gene expression following pTR-CBA-luciferase transfection (Fig. 5c). Further, inhibition of AAV transduction by Ionomycin was unaffected by vector dose (Fig. 5d). Taken together, these results suggest that regulation of calcium homeostasis in different cell types can significantly influence AAV transduction efficiency.

### Intracellular calcium affects early and late steps in the AAV infectious pathway.

**FIG 6:**
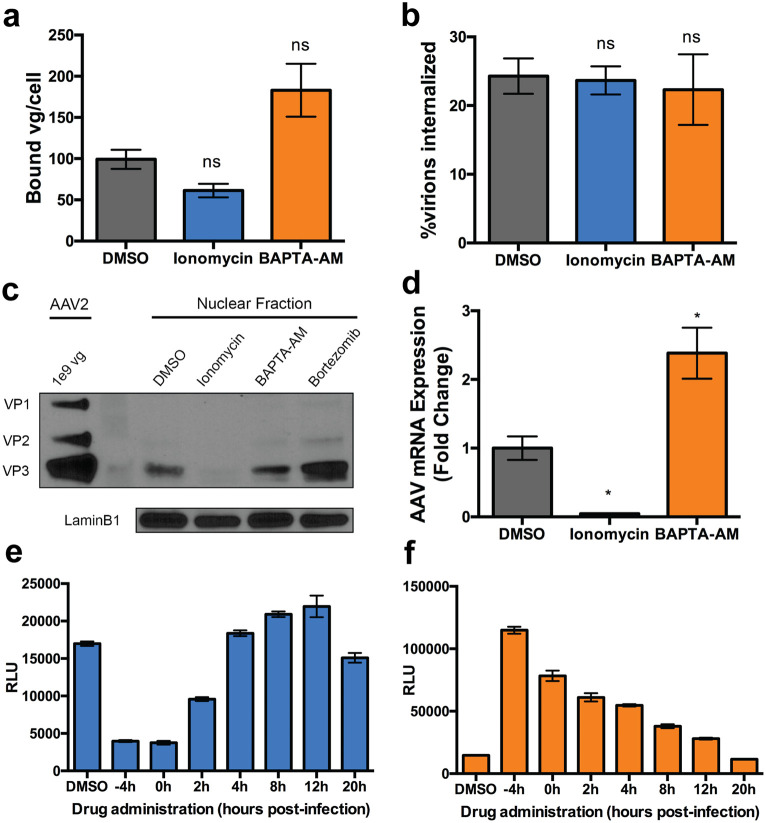
Interrogation of AAV trafficking steps with calcium modulating drugs. (a and b) Binding (a) and cellular uptake (b) of AAV2-luciferase. (c) Western blot with B1 (AAV capsid) and lamin B1 of nuclear extract from cells transduced with AAV2. (d) AAV2-luciferase transgene mRNA expression relative to the housekeeping gene. (e and f) Luciferase expression in HeLa cells treated with DMSO, ionomycin (e) or BAPTA-AM (f) at various times in relation to transduction with AAV2-Luc vectors. *, *P < *0.05; ns, not significant.

To understand how intracellular calcium influences AAV transduction, we sought to investigate several major steps in the AAV infectious pathway. It is worth noting that, due to the mechanism of action of ionomycin and BAPTA-AM, extracellular calcium in cell media is unaffected (19). Treatment with ionomycin or BAPTA-AM alters AAV2 capsid binding to the cell by approximately 2-fold (Fig. 6a). Although the effects are modest, it is likely that altering intracellular calcium levels can lead to altered composition of the outer leaflet of the plasma membrane, leading to increased viral binding. This observation could contribute to the changes in transduction efficiencies upon BAPTA-AM treatment discussed earlier. However, AAV internalization is not affected by either treatment in a synchronized infection (Fig. 6b).

Assessment of AAV capsid nuclear entry by Western blotting demonstrates that this trafficking step is markedly reduced (∼5-fold) upon treatment with ionomycin but not BAPTA-AM. As expected, an ∼2-fold increase in nuclear entry was observed upon treatment with the proteasome inhibitor bortezomib, included as a positive control (20) (Fig. 6c). In addition, ionomycin treatment decreased AAV vector genome mRNA levels by ∼20-fold (Fig. 6d), as expected due to the low number of vectors present in the nucleus (Fig. 6c). Further, mRNA levels from AAV delivered genomes increased by ∼2.5-fold upon BAPTA-AM treatment, concordant with the trend seen in transgene expression (Fig. 6d). Taken together, these data indicate that modulation of intracellular calcium by pharmacological agents can alter AAV trafficking and transduction.

### Ionomycin and BAPTA-AM affect AAV transduction through distinct kinetic mechanisms.

To dissect the differential impact of BAPTA-AM and ionomycin on AAV transduction, we carried out a time course study. As seen in Fig. 6e, the inhibitory effect of ionomycin is evident at 2 h after viral incubation. However, when added at 4 h postincubation or later, ionomycin no longer demonstrates an inhibitory effect. Conversely, BAPTA-AM increases transduction at every time point assessed up to 12 h postinfection, albeit at steadily decreasing effectiveness (Fig. 6f). These contrasting observations suggest that (i) ionomycin is unable to exert an inhibitory effect once viral entry into the nucleus has been initiated and (ii) BAPTA-AM plausibly operates through pleiotropic mechanisms on multiple intracellular events in the AAV infectious pathway.

### BAPTA-AM increases AAV transduction in the mouse brain.

**FIG 7:**
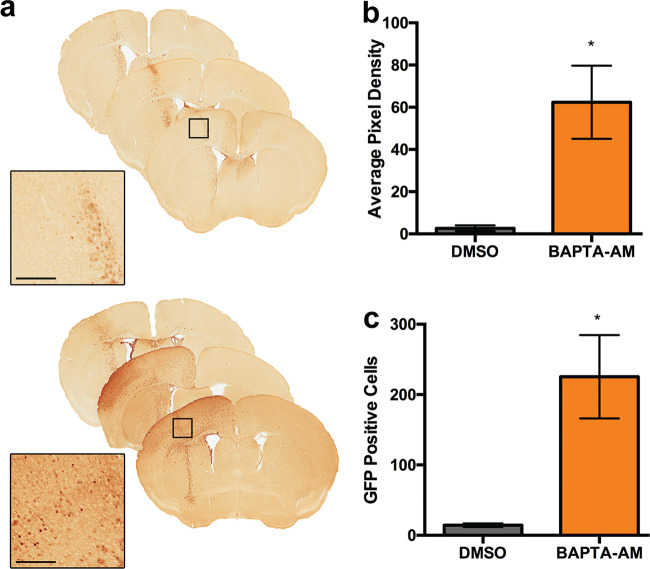
BAPTA-AM enhances AAV transduction in the mouse brain. (a) Bright-field scanned micrographs of immunohistochemically stained brain sections injected with AAV1 vectors packaging an scGFP transgene cassette in the presence of DMSO or BAPTA-AM. Black boxes indicate areas imaged at higher magnification. Bar = 300 μm. (b) Semiquantitative analysis of immunohistochemical signal. (c) Quantification of immunostained GFP-positive cells in the motor cortex. *, *P < *0.05.

Given the importance of calcium homeostasis in brain function and, correspondingly, calcium dysregulation in disease (21), we sought to determine whether modulating calcium *in vivo* can influence AAV transduction *in vivo*. Briefly, we directly injected AAV1 vectors packaging supercharged green fluorescent protein (scGFP) into the cerebrospinal fluid via intracerebroventricular (ICV) injection in neonatal C57/B6 mice. As seen in Fig. 7a, coadministration of BAPTA-AM with AAV1 increased transduction in the injected hemisphere. Quantification of GFP expression along the entire cortex (Fig. 7b) and the number of transduced neurons in the motor cortex (Fig. 7c) demonstrates a 15- to 20-fold increase in transduction upon BAPTA-AM treatment. These data demonstrate that pharmacological modulation of calcium levels in the brain can potentially help augment AAV transduction.

## DISCUSSION

In the current study, we investigated the role of *ATP2C1* and cellular calcium homeostasis in AAV biology and transduction. The Golgi compartment calcium ATPase pump has been shown to be an essential host factor for members of the families *Paramyxoviridae*, *Flaviviridae*, and *Togaviridae*. Specifically, SPCA1 (encoded by *ATP2C1*) appears to be exploited by diverse viruses, such as measles virus, dengue virus, Zika virus, and West Nile virus, for maturation and spread (22). As noted above, the *ATP2C1* gene was listed as a hit in a haploid genetic screen for essential AAV host factors (9). The functions of SPCA1 range from maintaining calcium homeostasis to protein sorting and Golgi compartment integrity (11). These prior studies provided the rationale and scientific premise for the current study investigating the role of cellular calcium in AAV infection.

We first observed that *ATP2C1* is critical for AAV transduction, regardless of serotype or vector copy numbers incubated *in vitro*, supporting the notion that AAV exploits cellular calcium during infection. Interestingly, we noted that the absence of *ATP2C1* did not impede binding, uptake, or nuclear entry, as evidenced by qPCR and confocal microscopy studies. Previous studies characterized AAV capsids with mutations in the VP1/VP2 basic regions (BR), which serve as putative nuclear localization signals. Mutagenesis of BR2 and BR3 produced virions with similar punctate A20 localization as well as reduced transduction (16). Further, mutagenesis of residues in the PLA2 domain and other VP1 regions also results in similar cytosolic A20 localization (8). While our study did not focus on capsid mutations, it is important to note that these capsid mutants utilized by other groups were in fact deficient in nuclear entry (8, 16). However, in the current study, nuclear entry was unaffected. Instead, the observed A20 immunostaining pattern suggests that intact virions do not traffic through the perinuclear Golgi network in *ATP2C1* KO cells relative to Scr controls. While the altered cytosolic trafficking pattern appears to be innocuous for AAV capsids entering the nucleus and uncoating, we observed a significant decrease in the efficiency of mRNA transcription from the AAV vector genome. Thus, the inability of AAV particles to traffic through the Golgi network prior to nuclear entry appears to correlate with a defect in genome transcription. These observations are corroborated by earlier reports that the AAV capsid is involved in regulating vector genome transcription (23–26).

Previous *in vitro* characterization of capsid conformation across pH changes has highlighted conformational changes during exposure to acidic environments or heat (7, 27, 28). Further, treatment of AAV capsids with trypsin generates smaller capsid protein cleavage products from VP1, VP2, and VP3 despite capsids remaining intact (29). In the current study, biochemical and immunofluorescent interrogation revealed defects in capsid conformational changes in *ATP2C1* KO cells. Further, *in vitro* capsid stability studies demonstrated that increased calcium can impede recognition of buried epitopes upon modest thermal treatment of AAV capsids. These findings are particularly interesting against the backdrop of recent studies that highlighted a role for the essential host factors AAVR and GPR108 in perinuclear Golgi compartment trafficking of AAV capsids (3, 7). Importantly, GPR108 has been postulated to engage with the VP1 N-terminal domain following exposure (7). Here, we show that calcium levels in the perinuclear Golgi region regulated by the calcium ATPase are critical for capsid conformational changes to occur. These latter events appear in turn to be essential for supporting mRNA transcription from AAV genomes.

**FIG 8:**
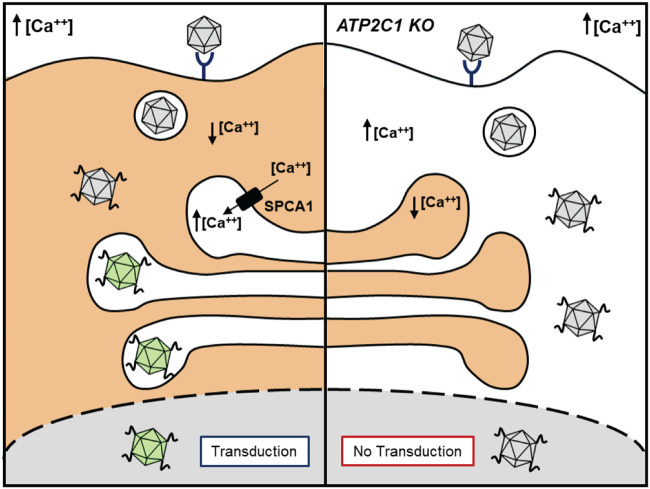
Schematic outlining the essential role of intracellular calcium homeostasis in AAV capsid dynamics and transduction. (Left) In wild-type cells, the calcium ATPase pump expression regulates the influx of cytosolic calcium into the Golgi compartment. The maintenance of a calcium gradient allows proper trafficking of AAV capsids to the perinuclear Golgi region, where the AAV capsids are “primed,” leading to productive transduction. (Right) In *ATP2C1* KO cells, where the calcium ATPase pump is not expressed, elevated cytosolic calcium levels inhibit AAV trafficking through the perinuclear compartment. Lack of processing within the Golgi compartment results in transduction-deficient AAV capsids.

Taken together, these observations corroborate that AAV capsid conformational changes are regulated by the intracellular calcium environment (Fig. 8). First, we postulate that following VP1/PLA2 domain externalization in low-pH compartments, such as endosomal vesicles, AAV capsids enter the high-calcium environment of the Golgi network maintained by SPCA1. In this calcium-rich perinuclear compartment, it is likely that AAV capsids undergo additional processing and conformational changes prior to nuclear entry. These conformational changes, in turn, appear to be critical for generating infectious AAV particles that support efficient transcription of the viral genome in the nucleus. As a corollary, disruption of the intracellular calcium gradient impedes perinuclear trafficking and Golgi compartment entry. While this aspect does not appear to preclude nuclear entry, the apparent lack of “priming” within the perinuclear compartment yields noninfectious AAV particles incapable of supporting genome transcription. This model is further supported by the finding that SPCA1 regulates calcium-dependent protease activity within the *trans*-Golgi network, which in turn is known to play a critical role in viral glycoprotein maturation for several viruses (18). For instance, reduction of Golgi compartment calcium levels is critical for enterovirus (e.g., polioviruses and coxsackieviruses) replication by inhibiting host intracellular protein trafficking pathways and blunting innate immune responses (30–32). These results highlight the important role of calcium in controlling viral protein processing and trafficking through the Golgi subcellular compartment.

From a pharmacological modulation perspective, while treatment of Scr and Huh7 cells with the calcium chelator BAPTA-AM modestly increased transduction, BAPTA-AM administration rescued AAV transduction in *ATP2C1* KO cells. Although speculative, given the ability of BAPTA-AM to chelate calcium, it is plausible that decreasing calcium levels in the cytosol of *ATP2C1* KO cells restores a calcium gradient promoting viral entry into the Golgi compartment and enables capsid conformational changes essential for efficient transduction. In contrast, allowing extracellular calcium to enter the cytosol and other organelles through pores generated by ionomycin treatment decreases AAV transduction. Interestingly, ionomycin severely inhibited nuclear accumulation of AAV, indicating that either nuclear entry or an upstream step is affected. It has been demonstrated in the past that ionomycin does not alter the nuclear pore complex or nuclear import of other substrates, suggesting that nuclear import might not be affected (33). Notably, ionomycin has no effect on transduction when added 4 h postransduction, suggesting a block potentially upstream of nuclear entry. This observation is distinct from those made with BAPTA-AM, which enhanced AAV transduction during multiple steps in AAV cell entry. Additional studies are required to assess calcium dynamics during AAV trafficking in order to tie the latter observations to the *ATP2C1* KO phenotype.

Last, we demonstrate that pharmacological modulation of calcium in the brain could offer a viable approach to augment AAV transduction. Calcium plays an important role in the central nervous system (CNS), having a primary role in the depolarization of neurons (21, 34). Additionally, prolonged disruption of calcium homeostasis in neurons has been shown to result in neuronal cell death (35). Furthermore, several neurological conditions have been linked to perturbations in calcium homeostasis in the CNS, such as Alzheimer’s disease and spinocerebellar ataxia (36–39). Notably, there are several approved drugs currently in use that safely alter calcium homeostasis in the CNS, e.g., benzodiazepines and dihydropyridines (40, 41). Thus, continued evaluation of calcium-modulating pharmacological agents as potential adjuvants or inhibitors of AAV transduction in the CNS is warranted. The study of calcium modulation *in vivo* could therefore help us better understand the impact of cellular calcium on AAV transduction in different cell and tissue types and the potential for pharmacological modulation to impact gene expression.

## MATERIALS AND METHODS

### Plasmids and reagents.

Guide RNAs against *ATP2C1* were designed and ordered as a single-stranded oligonucleotide from IDT (5′-GGAGCTGTCACCTTAGAACA-3′); the scrambled control guide (Scr; 5′-GGTCTCTGTACGGGCCGCCC-3′) does not align with the human genome by primer blast. Following phosphorylation and annealing, guides were ligated into BsmBI-digested LentiCrisprV2 (a gift from the Feng Zhang lab; Addgene plasmid number 52961). Mouse anti-actin (ab3280) was obtained from Abcam (Cambridge, MA), while goat-anti-mouse horseradish peroxidase (HRP) (32430) was obtained from Thermo Fisher. Rabbit anti-lamin B1 (12586) was obtained from Cell Signaling Technology (Danvers, MA). Hybridoma AAV antibodies A1, A69, and B1 were purchased from American Research Products, Inc. (Waltham, MA). Fluo4-am (no. F14201) was purchased from Thermo Fisher. Ionomycin (2092) and BAPTA-AM (2787) were obtained from Tocris Bioscience (Minneapolis, MN). Bortezomib (S1013) was obtained from Selleck Chemicals (Houston, TX).

### Recombinant virus production.

Recombinant AAV vectors packaging a chicken β-actin (CBA) promoter driving a firefly luciferase cassette as well as self-complementary AAV (scAAV) vectors packaging a hybrid CBA (CBh) promoter driving GFP were generated using triple plasmid transfection in HEK293 cells, as described previously (42). Viral titers were obtained as previously indicated, with viral preparations subjected to DNase (10 mg/ml) treatment to remove unencapsidated viral genomes, followed by DNase inactivation with 0.5 M EDTA and proteinase K digestion to release viral genomes from capsids (42). Viral DNA was quantified using quantitative PCR comparing samples against vector core standards, using primers against the ITRs. qPCR was performed using a Roche LightCycler 480 (Roche Applied Sciences, Pleasanton, CA).

Pseudotyped recombinant lentivirus packaging guides against *ATP2C1* or Scr control guides were produced by triple plasmid transfection of psPax2, vesicular stomatitis virus G protein (VSVG), and the LentiCrisprV2 packaging cassette into HEK293s, as previously indicated (43). Medium supernatant containing recombinant lentivirus was harvested 48 h posttransfection and filtered before lentiviral transduction.

### Tissue culture and cell line generation.

Huh7 hepatocarcinoma cells were obtained from the University of North Carolina at Chapel Hill Lineberger Tissue Culture Facility, while HEK293 human embryonic kidney cells were obtained from the University of North Carolina at Chapel Hill Vector Core. Cells were maintained in Dulbecco’s modified Eagle’s medium (DMEM), with supplementation of 100 U/ml penicillin, 100 μg/ml streptomycin, and 10% fetal bovine serum (FBS). Cells were kept in 5% CO_2_ at 37°C. Stable scramble guide (Scr) and *ATP2C1* KO cell lines were generated via spinoculation, where cells were seeded at 1.5 × 10^5^ cells/well in a 6-well plate and incubated with medium containing lentivirus and 8 μg/ml Polybrene for 15 min. These plates were then spinoculated for 30 min at 400 × *g* at room temperature and incubated for 2 to 4 h at 37°C after spinfection, after which medium was replaced with medium containing lentivirus without Polybrene. At 48 h after spinoculation, cells were subjected to puromycin selection. After 1 week of antibiotic selection, single clones were generated via serial dilution. The indel mutation rate of serial clones was determined via high-throughput sequencing of an amplicon surrounding the *ATP2C1* guide site. Briefly, genomic DNA was harvested from Scr and *ATP2C1* KO Huh7 cells and subjected to PCR amplification (forward primer, 5′-GTGGCTAATCAATGGAAAAGGGA-3′; reverse primer, 5′-GGACACAGATTCTCCCCAGTTT-3′). PCR products were then gel extracted and subjected to AmpliconEZ sequencing analysis by Genewiz (Morrisville, NC).

### Luciferase transduction assays.

Cells were counted and seeded at 3 × 10^4^ cells/well in 24-well plates and allowed to adhere overnight. Cells were then transduced with AAV-CBA-luciferase vectors at various doses. At 24 h postransduction, cells were harvested in passive lysis buffer, and lysate was combined with luciferin substrate (Promega, Madison, WI). A VictorX plate reader was used to quantify luciferase signal (PerkinElmer, Waltham, MA).

### Binding, uptake, and nuclear uptake studies.

For binding studies, Scr and *ATP2C1* KO Huh7 cells were seeded at 5 × 10^4^ cells/well in 12-well plates and allowed to settle overnight. Cells were then prechilled at 4°C for 30 min and then treated with recombinant AAV (rAAV)-CBA-luciferase at 4°C for 1 h, followed by three washes with ice-cold 1× phosphate-buffered saline (1× PBS). Double-distilled water (300 μl) was then added to each well, and following three freeze-thaw cycles, genomic DNA was extracted using the IBI mini-genomic DNA kit (IBI, Dubuque, IA).

For cellular uptake studies, following removal of unbound virions, cells were immersed in warm complete medium and transferred to a 37°C incubator to synchronize viral internalization. After 1 h of incubation, medium was removed, and cells were treated with 0.05% trypsin to dissociate cell-surface associated virions. Following quenching of trypsin with complete medium, cells were transferred to Eppendorf tubes and subjected to three additional washes with cold 1× PBS. Total genomic DNA was then extracted as described above. Quantification of viral genomes per cell for both binding and uptake was determined via qPCR of DNA samples with primers against the luciferase transgene and the host laminin gene, as previously described (42).

For interrogation of nuclear uptake, Scr and *ATP2C1* KO cells were seeded at 1.5 × 10^5^ cells per well, allowed to settle overnight, and then transduced with rAAV2-luciferase. Cells were trypsinized, harvested as a pellet, and washed with 1× PBS, after which cytoplasmic and nuclear fractions were harvested with the NE-PER kit following the manufacturer’s instructions (Thermo Fisher). Genomic DNA was purified from fractions as described above, and then fractions were subjected to qPCR as indicated above to quantify vector genomes per cell fraction.

### Quantitation of transcribed mRNA from AAV vector genomes.

Scr and ATP2C1 KO cells were plated in a 24-well plate at 3 × 10^4^/well and allowed to settle overnight. Cells were infected at 2 × 10^3^ viral genomes (vg)/cell with AAV2 packaging a CBA-luciferase genome. At 24 h postinfection, cells were harvested with an RNeasy Plus minikit according to the manufacturer’s protocol (catalog number 74134; Qiagen). cDNA was generated from total RNA via RT-PCR (catalog number 4388950; Thermo Fisher). cDNA samples were then analyzed with qPCR using primers against AAV luciferase mRNA (forward, 5′-AAAAGCACTCTGATTGACAAATAC-3′; reverse, 5′-CCTTCGCTTCAAAAAATGGAAC-3′) and the LAMB1 housekeeping gene (forward, 5′-GTTAACAGTCAGGCGCATGGGCC-3′; reverse, 5′-CCATCAGGGTCACCTCTGGTTCC-3′). Fold change mRNA was determined with the ΔΔ*C_T_* method.

### Western blotting, dot blotting, and immunoprecipitation studies.

For protein expression analysis of ATP2C1, both Scr and *ATP2C1* KO cell pellets were lysed in radioimmunoprecipitation assay (RIPA) buffer with 1× Halt protease inhibitor (Thermo Fisher) for 45 min at 4°C. Lysates were spun at maximum speed for 10 min at 4°C to remove cellular debris. Lithium dodecyl sulfate (LDS) sample buffer (1×) with 10 mM dithiothreitol (DTT) was added to cleared lysates and boiled for 2 min. Samples were then analyzed via SDS-PAGE (NuPAGE 4 to 12% bis-Tris gel) and transferred onto nitrocellulose membrane (Thermo Scientific). Following blocking in 5% milk–1× Tris-buffered saline containing Tween 20 (TBST), samples were incubated with primary antibodies at either 1:1,000 (ATP2C1; Abnova 2G1) or 1:2,000 (actin) overnight in 5% milk–1× TBST. Following three 1× TBST washes, samples were incubated with secondary antibody conjugated to HRP at 1:20,000 in 5% milk–1× TBST for 1 h. The signal was the visualized via SuperSignal West Femto maximum-sensitivity substrate (Thermo Scientific) according to the manufacturer’s instructions.

For immunoprecipitation studies, Scr and *ATP2C1* KO cells were incubated with 20,000 vg/cell AAV2-luciferase for 18 h, then washed with ice-cold 1× PBS, and harvested in RIPA buffer with 1× Halt protease inhibitor (Thermo Fisher) for 30 min on ice. Lysates were spun at maximum speed for 10 min at 4°C to remove cellular debris, and then supernatants were subject to preclearing by 1 h incubation with 25 μl protein G magnetic beads (GE) on a nutator at 4°C. A1 and A69 hybridoma medium antibodies (250 μl) (14, 15) were bound to 25 μl protein G magnetic beads for 1 h at 4°C, after which antibody supernatant was removed and precleared lysate was loaded onto antibody-bound beads. Immunoprecipitations were then carried out overnight on a nutator at 4°C, and the mixtures were then eluted in 10 mM DTT and 1× LDS buffer for 10 min at 95°C. Samples were then analyzed via SDS-PAGE (NuPAGE 4 to 12% bis-Tris gel) and transferred onto nitrocellulose membranes (Thermo Scientific). Following blocking in 5% milk–1× TBST, samples were incubated with primary antibodies at either 1:2,000 (actin) or 1:20 (B1) for 1 h in 2% milk–1× TBST. Following three 1× TBST washes, samples were incubated with secondary antibody conjugated to HRP at 1:20,000 in 2% milk–1× TBST for 1 h. The signal was the visualized via SuperSignal West Femto maximum-sensitivity substrate (Thermo Scientific) according to the manufacturer’s instructions.

For capsid dot blots, a thermal cycler was used to subject AAV2-CBA-Luc to heat treatment. The virus was combined with either 5 mM EGTA, 2 mM CaCl_2_, or both in PBS (without CaCl_2_) in a total volume of 200 μl and then heated to temperatures ranging from 40°C to 70°C, while including a 95°C control, for 30 min. The viruses were then cooled to 4°C before being loaded onto a nitrocellulose membrane in a dot blot apparatus. The membranes were blocked with 5% milk–1× TBST and then blotted with antibody B1 or A1 at a dilution of 1:30 in 2% milk–1× TBST for 1 h followed by HRP-conjugated goat anti-mouse antibody at a dilution of 1:10,000 for 1 h. The blots were then washed 3 times with 1× TBST for 15 min each, followed by detection using the SuperSignal West Femto kit (Thermo Fisher, Waltham, MA).

### Fluorescence microscopy studies.

Scramble and ATP2C1 KO cells were seeded on coverslips in 24-well plates at a density of 5 × 10^4^ cells/well and allowed to adhere overnight. Cells were transduced with AAV2 packaging CBA-luciferase for 18 h. After 1× PBS washes, the cells were fixed in 4% paraformaldehyde, permeabilized with 0.1% Triton X-100, and blocked in 5% normal goat serum (NGS) diluted in 1× PBS. A20 hybridoma antibody and golgin-97 antibody were diluted 1:10 and 1:100 in 5% NGS in 1× PBS, respectively. For assessing capsid conformational changes, the A69 mouse hybridoma antibody was utilized. After the cells had been incubated with the antibodies for 1 h, the cells were washed three times with 1× PBS. Fluorescent secondary antibodies were used to stain the cells for 1 h at a dilution of 1:400. All samples were stained with DAPI (4′,6-diamidino-2-phenylindole) prior to mounting in Prolong Diamond (Invitrogen). Samples were imaged using an ECHO Revolve microscope. ImageJ was used to adjust brightness and contrast equally across data sets.

### Animal studies.

Animal experiments were carried out with C57/B6 mice bred and maintained in accordance with NIH guidelines and as approved by the UNC Institutional Animal Care and Use Committee (IACUC). Intracerebroventricular (ICV) injections were performed as described previously (44). Briefly, neonatal postnatal day 0 (P0) pups were rapidly anesthetized by hypothermia for 1 min followed by stereotaxic intraventricular cerebral injections. A Hamilton 700 series syringe with a 26-gauge needle (Sigma-Aldrich) was attached to a model 900 small animal stereotaxic instrument (Kopf, Tujunga, CA). Mice were injected in the left lateral ventricle with 2 × 10^9^ vector genome-containing particles of self-complementary AAV1-CBh-GFP with either vehicle control or BAPTA-AM in a total volume of 3 μl. Mouse brains were harvested 14 days postinjection.

### Sectioning, immunostaining, and analysis of brain tissue.

After postfixation in 4% paraformaldehyde (PFA), brains were sectioned into 50-μm sections using a Leica VT1200S vibrating-blade microtome. Brains were then blocked and permeabilized in 10% normal goat serum and 1% Triton X-100 in 1× PBS for 1 h at room temperature. Primary antibody staining was performed with mouse anti-GFP antibody overnight at 4°C. Brains were then stained with 3,3′-diaminobenzidine (DAB) using a Vectastain ABC peroxidase kit (PK-4001) from Vector Laboratories (Burlingame, CA) according to the manufacturer’s instructions. Development of the stained tissues was carried out using the DAB staining kit (D7304) from Sigma-Aldrich (St. Louis, MO). Slides were imaged by the UNC at Chapel Hill Translational Pathology Laboratory using the Aperio ScanScope XT system. Images were analyzed using the Aperio ImageScope software, and quantification was performed using ImageJ software.

### Statistical analyses.

Data are expressed as means with standard errors. A two-tailed unpaired Student's *t* test was used and calculated with GraphPad Prism version 6. *P* values below 0.05 were considered significant.
